# Endoscopic Calcaneoplasty Achieves Excellent Functional Outcomes in Haglund's Deformity

**DOI:** 10.1002/ars2.70108

**Published:** 2026-09-25

**Authors:** Samuel Sing Li Ong, Raj Kumar Socklingam, Ing How Moo, Charles Kam King Kon

**Affiliations:** ^1^ Department of Orthopaedic Surgery Changi General Hospital Singapore Singapore

## Abstract

**Purpose:**

To evaluate the functional outcomes of endoscopic calcaneoplasty for patients with Haglund's deformity who are recalcitrant to conservative management and to assess complication rates and the proportion of patients achieving minimal clinically important difference in functional outcome scores.

**Methods:**

Patients with Haglund's deformity who were treated at our tertiary hospital between July 2020 and October 2024 were evaluated for inclusion. Endoscopic calcaneoplasty was performed with the patient positioned supine. Functional outcomes were assessed using the visual analog scale (VAS), American Orthopaedic Foot and Ankle Society (AOFAS) ankle‐hindfoot score, and Foot Function Index (FFI) score preoperatively and postoperatively at final follow‐up, with a minimum follow‐up of 15 months.

**Results:**

The mean follow‐up period was 32.3 months (range, 15‐66 months). A total of 68 cases (59 patients, 69 feet) were analyzed for this study. The overall mean age was 54.7 ± 13.8 years. 47.1% were men, whereas 52.9% were women. Preoperatively, patients reported that their mean VAS score was 7.8 ± 1.6, which markedly diminished to 1.2 ± 2.0 postoperatively. The baseline mean AOFAS score was 56.0 ± 13.1, and this improved to 92.1 ± 11.7 after surgery. The mean FFI score also significantly improved from 55.0 ± 23.2 preoperatively to 10.8 ± 18.2 postoperatively. All 3 outcome measures showed statistically significant differences (*P* < .001). Sex‐stratified analysis showed no statistically significant differences across all 3 outcome scores (all *P* > .05). Most patients achieved the minimal clinically important difference, with 98.5% for VAS, 97.1% for AOFAS, and 89.7% for FFI.

**Conclusions:**

In patients with Haglund's deformity, treatment with endoscopic calcaneoplasty in a supine position showed consistent and significant postoperative improvements in VAS, AOFAS ankle‐hindfoot score, and FFI score, with a low complication rate at a minimum 15‐month follow‐up.

**Level of Evidence:**

Level IV, therapeutic case series.

## Graphical abstract

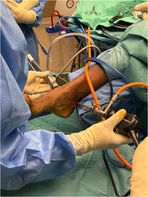

Haglund's deformity, also commonly referred to as a “pump bump,” was first described in the literature by the Swedish orthopaedic surgeon Patrick Haglund in 1928.^1^ It is characterized by an abnormal bony exostosis on the posterior‐superior aspect of the calcaneum and is a leading cause of posterior heel pain. In the United Kingdom, it has been reported that 1 in 8 individuals aged 50 years or older experience posterior heel pain and over half of them describe it as disabling.^2^

In addition to posterior heel pain, patients may also complain of a noticeable lump, swelling, erythema, or warmth over the back of the heel. These symptoms are often exacerbated by inappropriate footwear and physical activities.^3^ A Haglund's deformity can be identified as posterior‐superior calcaneal exostosis on plain radiographs of the lateral ankle or foot.^4^

This condition is frequently associated with wearing low‐back shoes, which results in mechanical irritation of the heel, culminating in insertional Achilles tendinopathy and retrocalcaneal bursitis.^5^ Although the precise cause remains unclear, proposed risk factors include obesity, manual vocations, physical inactivity, pes cavus, a varus heel, and a tight Achilles tendon.^2^, ^6^

The treatment options for Haglund's deformity can be broadly categorized into conservative and surgical management. Conservative management includes nonsteroidal anti‐inflammatory drugs, physiotherapy (especially eccentric exercises), heel lifts, heel pads, footwear modifications (open back shoes), and orthotic devices.^7^ If a trial of conservative management fails for at least 3 to 6 months, surgical intervention may be warranted.^4^

Traditional surgical options include open calcaneoplasty with or without retrocalcaneal bursectomy, Achilles tendon debridement, detachment, or attachment. This can be performed via a central tendon splitting, medial, or lateral approach. An alternative technique is a dorsal closing wedge calcaneal osteotomy (also known as a Zadek osteotomy),^8^ which can be done open or percutaneously.

First described in the literature by van Dijk et al. in 2001, endoscopic calcaneoplasty has gained popularity because it offers the advantages of smaller incisions, reduced morbidity, and a faster recovery process.^9^ It also provides surgeons an excellent direct visualization of the retrocalcaneal anatomy. For this study, we explore an endoscopic calcaneoplasty technique in which the procedure is performed with the patient in the supine position, deviating from the conventional prone position as originally proposed by van Dijk.^9^

The purposes of this study were to evaluate the functional outcomes of endoscopic calcaneoplasty for patients with Haglund's deformity who are recalcitrant to conservative management and to assess complication rates and the proportion of patients achieving minimal clinically important difference (MCID) in functional outcome scores. We hypothesized that endoscopic calcaneoplasty will offer significant benefits in terms of pain relief and a swift return to function while maintaining a low complication rate.

## METHODS

This is a retrospective functional outcome study conducted at a tertiary hospital on patients with Haglund's deformity who were treated between July 2020 and October 2024. Ethical approval was secured from the Institutional Review Board—Ethics and Compliance Online System (reference 2020‐2642). Eligible patients provided informed consent in accordance with our local guidelines.

### Participants

Consecutive patients who had undergone endoscopic calcaneoplasty during the study period were screened for eligibility. The inclusion criteria included patients who had symptomatic Haglund's deformity for 3 or more months refractory to nonoperative management, ≥21 years of age, and given informed consent to participate in the study. The exclusion criteria were patients who presented with multiple foot pathologies besides Haglund's deformity, a history of calcaneal fractures, a history of previous Achilles tendon repair or reconstruction surgery, pregnancy, a history or risk of venous thromboembolic events, and neuromuscular disorders affecting foot biomechanics.

The diagnosis of Haglund's deformity was made by a senior foot and ankle orthopaedic surgeon (C.K.K.K.) based on a complete clinical history and examination and plain radiographs of the lateral ankle.

Endoscopic calcaneoplasty was indicated in patients with persistent posterior heel pain because of Haglund's deformity, and they had attempted at least 3 months of nonoperative management, including analgesia, physiotherapy, and footwear modifications. The mean duration of symptoms experienced was defined as the interval between the patient‐reported onset of symptoms and the date of surgery.

### Surgical Technique

**FIGURE 1:**
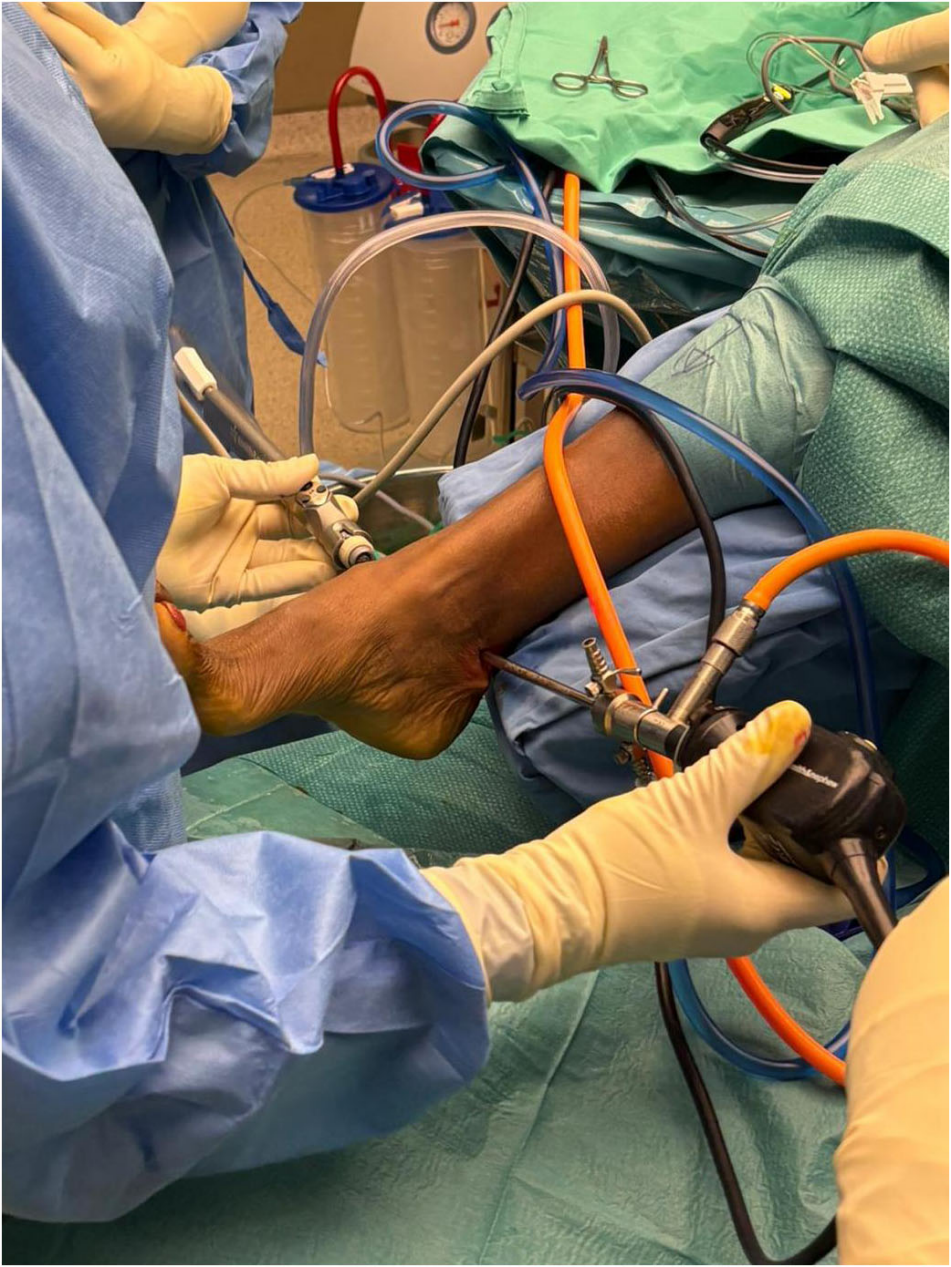
Surgical setup with patient in a supine position and a triangular cushion placed below the knee with medial and lateral portals established.

**FIGURE 2:**
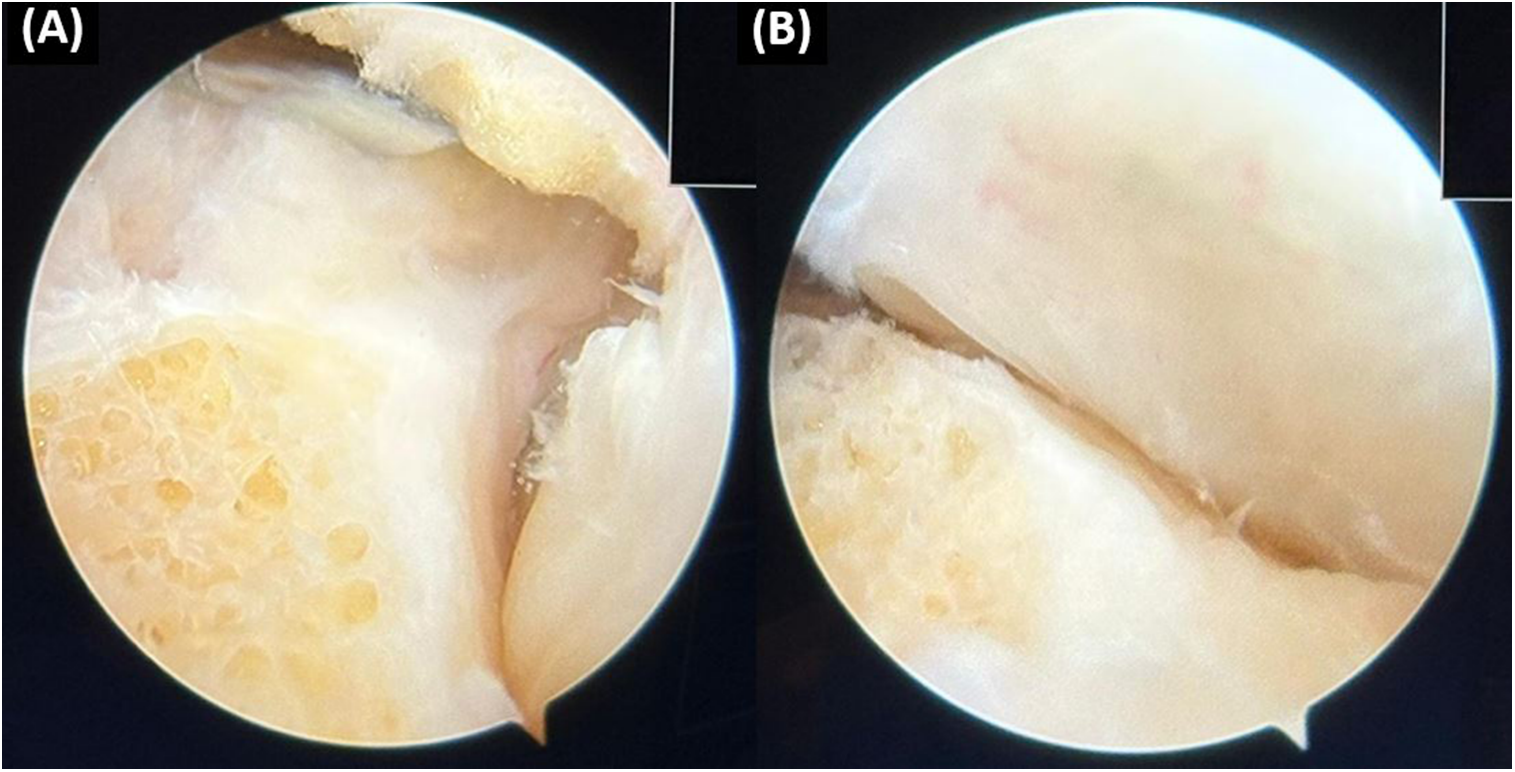
Intraoperative images of endoscopic calcaneoplasty for Haglund's deformity, (A) preresection and (B) postresection.

Endoscopic calcaneoplasty was performed as per the technique paper published by Koh et al.^10^ The patient is positioned supine with a triangular cushion placed below the knee to allow flexion of both the hip and knee joints (Figure 1). As a result, the operated ankle is passively held in a gravity‐assisted plantarflexed position. Medial and lateral portals are created to facilitate soft‐tissue debridement, after which bony resection is performed distally to proximally in close proximity to the Achilles tendon (Figure 2). The ankle can be further plantarflexed to increase the angle between the Achilles tendon and the superior calcaneal tuberosity to create more space for bony resection. Postoperatively, the patients are allowed to full weight‐bear and start active range‐of‐motion exercises immediately.

### Outcome Measures

The visual analog scale (VAS), American Orthopaedic Foot and Ankle Society (AOFAS) ankle‐hindfoot score, and Foot Function Index (FFI) score were measured preoperatively and postoperatively at the date of their last follow‐up. VAS was measured at rest.

### Statistical Analysis

No formal sample size calculation was performed because this was a retrospective study, including all eligible patients during the study period.

For demographical data, it was summarized as mean ± standard deviation for numerical data and as frequency (N) with percentage for categorical data such as sex, laterality, and smoking status. Outcome measurement scores (VAS, AOFAS, and FFI) were displayed in mean ± standard deviation. A paired *t* test was performed to compare the preoperative and postoperative outcome scores. Normality of paired differences was assessed using visual inspection (histograms and Q‐Q plots). A 2‐tailed *P* < .05 was considered statistically significant.

Sex‐stratified analyses were performed in accordance with the Sex and Gender Equity in Research guidelines to evaluate differences between male and female patients. Between‐sex comparisons of changes in outcome scores were conducted using independent‐samples *t* tests. MCID analysis was calculated for the cohort using a distribution‐based approach, with the threshold defined as one‐half of the standard deviation of the change in the respective VAS, AOFAS, and FFI scores.

Statistical analysis was performed using IBM SPSS Statistics for Windows, Version 31.0 (IBM, Armonk, NY).

## RESULTS

### Patient Demographics

**FIGURE 3:**
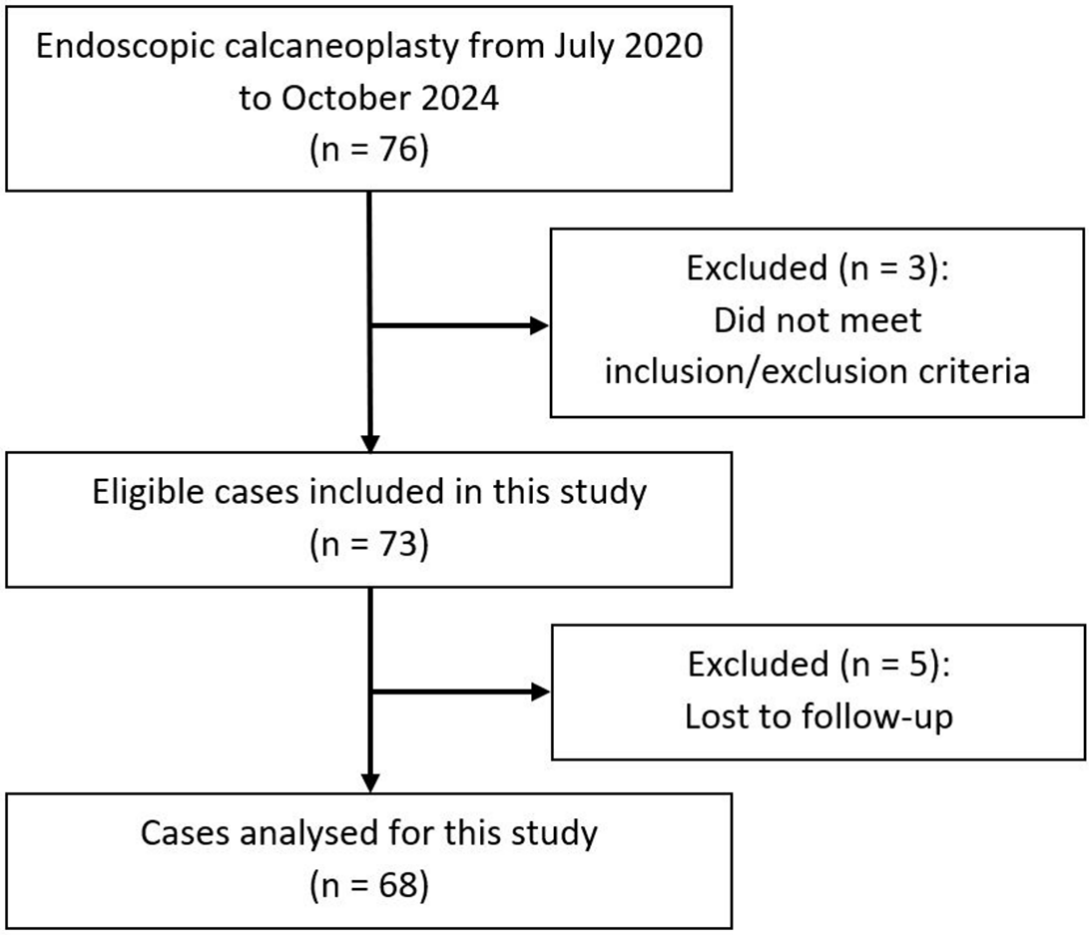
Patient flowchart.

Seventy‐six cases of endoscopic calcaneoplasty were performed during our study period. After assessment for eligibility, 73 cases were included in this study. However, out of the 73 cases selected, 5 patients were lost to follow‐up. Hence, a total of 68 cases (59 patients, 69 feet) were analyzed for this functional study (Figure 3).

Table 1 shows the demographics of the 68 cases having an overall mean age of 54.7 ± 13.8 years with a range of 23 to 80 years of age. Among these cases, 32 were men (47.1%), whereas 36 were women (52.9%). Endoscopic calcaneoplasty was performed on the right in 32 cases (47.1%) and on the left in 35 cases (51.5%), and 1 patient had bilateral surgery done in the same setting (1.5%). Of note, 8 patients had staged bilateral surgery with a mean of 13.1 months between procedures. The mean body mass index was 30.0 ± 5.78. The mean duration of symptoms experienced was 26.0 ± 38.6 months, defined as the interval between the patient‐reported onset of symptoms and the date of surgery. Most were nonsmokers (61 cases, 89.7%), whereas 7 cases were smokers (10.3%). For diabetic status, 18 were diabetic (26.5%), whereas 50 were nondiabetic (73.5%).

**TABLE 1:** Patient Demographics

**Demographics**	**Mean ± SD (Range) or n (%)**
Age, yr	54.7 ± 13.8
Sex	
Male	32 (47.1%)
Female	36 (52.9%)
Laterality	
Right	32 (47.1%)
Left	35 (51.5%)
Bilateral	1 (1.5%)
BMI	30.0 ± 5.78
Duration of symptoms, mo	26.0 ± 38.6
Smoking status	
Yes	7 (10.3%)
No	61 (89.7%)
Diabetic	
Yes	18 (26.5%)
No	50 (73.5%)
Surgical type	
Primary	66 (97.1%)
Revision	2 (2.9%)

*Note*: Age, BMI, and duration of symptoms were presented in mean ± SD. Sex, laterality, smoking status, diabetic status, and primary/revision surgery were presented in numbers, N (%).

BMI, body mass index; mo, months; SD, standard deviation; yr, years.

Of the procedures performed, 66 cases were primary surgeries (97.1%), whereas 2 cases were revision surgeries (2.9%) in patients who had previously undergone surgical treatment for their Haglund's deformity. Among the revision cases, 1 had their primary surgery performed by another surgeon 6 years prior, whereas the other had their primary procedure performed by us and subsequently required revision surgery 15 months later.

### VAS, AOFAS, and FFI

The mean follow‐up period was 32.3 months (range, 15‐66 months). Table 2 presents the mean VAS, AOFAS, and FFI scores before and after endoscopic calcaneoplasty. The postoperative score was taken at the last follow‐up session.

**TABLE 2:** Mean VAS, AOFAS, and FFI Scores Comparing Preoperative and Postoperative

	**Preoperative (Mean ± SD)**	**Postoperative (Mean ± SD)**	**Mean Change (95% CI)**	***P*** **Value**
VAS	7.8 ± 1.6	1.2 ± 2.0	−6.7 (−7.3 to −6.1)	<.001
AOFAS	56.0 ± 13.1	92.1 ± 11.7	36.1 (32.4‐39.9)	<.001
FFI	55.0 ± 23.2	10.8 ± 18.2	−44.2 (−50.2 to −38.2)	<.001

*Note*: Mean is represented as mean ± SD with respective *P* value. Mean change calculated as postoperative score minus preoperative score. Negative values indicate improvement for VAS and FFI, whereas positive values indicate improvement for AOFAS. Paired *t* test was used for all comparisons. *P* value is considered significant if *P* < .05.

AOFAS, American Orthopaedic Foot and Ankle Society; CI, confidence interval; FFI, Foot Function Index; SD, standard deviation; VAS, visual analog scale.

Preoperatively, our patients reported their mean VAS score of 7.8 ± 1.6, which markedly diminished to 1.2 ± 2.0 postoperatively. The mean AOFAS score was 56.0 ± 13.1 at baseline, which improved to 92.1 ± 11.7 after surgery. The mean FFI score also showed significant improvement from 55.0 ± 23.2 preoperatively to 10.8 ± 18.2 postoperatively. All 3 outcome measures (VAS, AOFAS, and FFI) showed statistically significant difference from preoperative and postoperative assessments (*P* < .001).

At their last follow‐up session, 44 cases achieved a VAS score of 0, 33 cases obtained a perfect AOFAS ankle‐hindfoot score of 100, whereas 28 cases attained an FFI score of 0.

### Sex‐Based Analysis of Functional Outcomes

Functional outcomes were further stratified by sex in accordance with the Sex and Gender Equity in Research guidelines (Table 3). Both male and female groups showed statistically significant improvements in postoperative VAS, AOFAS, and FFI scores compared with their respective preoperative scores. Comparing men to women, there were no statistically significant differences in mean change for VAS, AOFAS, or FFI score (all *P* > .05), indicating that sex did not impact the magnitude of postoperative improvement.

**TABLE 3:** Mean VAS, AOFAS, and FFI Scores Comparing Preoperative and Postoperative in Men and Women

		**Preoperative (Mean ± SD)**	**Postoperative (Mean ± SD)**	**Mean Change (95% CI)**	***P*** **Value (Within Sex)**	***P*** **Value (Men vs Women)**
VAS	Male	7.5 ± 1.5	1.0 ± 1.7	−6.5 (−7.2 to −5.8)	<.001	.470
	Female	8.2 ± 1.7	1.3 ± 2.2	−6.9 (−7.9 to −6.0)	<.001	
AOFAS	Male	59.2 ± 13.3	95.0 ± 7.0	35.8 (30.4‐41.1)	<.001	.858
	Female	53.2 ± 12.4	89.6 ± 14.3	36.4 (31.0‐41.9)	<.001	
FFI	Male	45.4 ± 21.7	4.4 ± 5.5	−41.0 (−49.2 to −32.8)	<.001	.330
	Female	63.5 ± 21.3	16.6 ± 23.1	−46.9 (−55.9 to −38.0)	<.001	

*Note*: Within‐group pre‐ and postoperative comparisons were performed using paired‐samples *t* tests. Between‐sex comparisons of mean change in scores were performed using independent *t* tests. *P* value is considered significant if *P* < .05.

AOFAS, American Orthopaedic Foot and Ankle Society; CI, confidence interval; FFI, Foot Function Index; SD, standard deviation; VAS, visual analog scale.

### MCID Analysis

In Table 4, MCID thresholds were 1.2 for VAS, 7.7 for AOFAS, and 12.4 for FFI. A total of 98.5% of cases achieved MCID for VAS, 97.1% of AOFAS, and 89.7% for FFI.

**TABLE 4:** MCID analysis for VAS, AOFAS, and FFI Scores

	**Mean Change ± SD**	**MCID Threshold**	**% Achieving MCID**
VAS	6.7 ± 2.4	1.2	98.5
AOFAS	36.1 ± 15.4	7.7	97.1
FFI	44.2 ± 24.8	12.4	89.7

AOFAS, American Orthopaedic Foot and Ankle Society; FFI, Foot Function Index; MCID, minimal clinically important difference; SD, standard deviation; VAS, visual analog scale.

### Adverse Events

One patient underwent revision calcaneoplasty with Topaz radiofrequency treatment 15 months after her initial surgery performed under our care. This was because of persistent moderate heel pain attributable to inadequate bony resection, resulting in impingement and marked scar tissue formation. Another patient developed a neuroma over the calcaneal branch of the plantar nerve, which improved symptomatically after a trial of conservative management. Notably, there were no wound‐related complications observed in our study.

## DISCUSSION

This functional outcome study showed excellent results across all 3 scores (VAS, AOFAS, and FFI scores) after endoscopic calcaneoplasty, with statistically significant improvements in pain and function across all outcome measures. Moreover, most patients also achieved the MCID, indicating that these improvements were not only statistically significant but also clinically meaningful. Therefore, this paper contributes to the growing body of evidence supporting endoscopic calcaneoplasty as an efficacious alternative to open techniques for the treatment of Haglund's deformity.

Haglund's deformity is a frequently under‐recognized cause of debilitating posterior heel pain. Although conservative management is advocated as the first‐line treatment, it is only effective in approximately 60% to 80% of the cases.^11^ Hence, surgical intervention may be considered for those who do not experience adequate relief with nonoperative measures.

Surgery for Haglund's deformity can be performed using either open or endoscopic techniques. However, current systematic reviews and meta‐analysis do not provide strong evidence favoring 1 approach over the other. Still, endoscopic surgery has the advantages of shorter operating duration, better cosmesis, and lower complication rates.^12^

This study investigated the functional outcomes of endoscopic calcaneoplasty for Haglund's deformity in a supine position. This technique offers an ergonomic advantage to the operating surgeon and avoids position‐related risks with the prone position, such as pressure‐related injuries, anesthetic airway challenges, and positioning difficulties.^10^

In the existing literature, a study by Mahmoud et al.^13^ involving 17 patients (21 feet) with a minimum follow‐up of 4 years produced comparable results to those of our study with improvements of VAS score from a preoperative mean of 8.1 ± 1.4 to a postoperative mean of 0.7 ± 1.04 and AOFAS score which increased from 55.7 ± 9.3 to 94.3 ± 7.1. The author performed endoscopic calcaneoplasty using a biportal technique with the patient in the prone position, with the foot dangling over the table edge. Recently, Kapoor et al.^14^ conducted a prospective observational study of 30 patients who underwent endoscopic calcaneoplasty in the prone position. At 1‐year follow‐up, the VAS score decreased from 7.2 to 0.9, whereas the AOFAS score increased from 62.3 to 96.8. Moreover, Cusumano et al.^15^ examined 26 heels that underwent endoscopic calcaneoplasty, which showed substantial improvements in VAS score from 7.6 ± 1.3 to 1.3 ± 1.9 and AOFAS score from 66.69 ± 7.19 to 93.69 ± 10.04 and reduction in FFI (Pain) from 55.9 ± 12.3 to 9.6 ± 16.5 and FFI (Disability) from 48.7 ± 12.9 to 7.3 ± 12.8. Collectively, these 3 studies^13^, ^14^, ^15^ reported no major complications postoperatively, which reinforces endoscopic calcaneoplasty as a safe and viable alternative with good functional outcomes.

Comparing open and endoscopic approaches, a multicenter prospective study by Thiounn et al.^16^ showed that endoscopic calcaneoplasty resulted in a faster recovery, but both approaches yielded similar complication rates and identical outcomes in the long run. Cusumano et al.^14^ also reported no significant differences in VAS, AOFAS, and FFI scores between the 2 groups. Regarding complications, Wiegerinck et al.^17^ reported major complications (e.g., tendon rupture or deep infection) in 4.3% and minor complications (e.g., superficial skin infection or scar tenderness) in 45% after open surgery. The incidence decreased in the endoscopic group, with major complications in 0.7% and minor complications in 1.3%.

### Limitations

This study has several limitations. First, there was a lack of a control group to compare the outcomes of endoscopic calcaneoplasty to other surgical techniques (such as open surgery or alternative endoscopic methods). Second, radiological parameters on plain radiographs such as the Fowler‐Phillip angle and Heneghan‐Pavlov parallel pitch lines were not included in this functional outcome study. Finally, no preoperative or postoperative advanced imaging (magnetic resonance imaging) was performed to assess radiological changes.

## CONCLUSIONS

In patients with Haglund's deformity, treatment with endoscopic calcaneoplasty in a supine position showed consistent and significant postoperative improvements in VAS, AOFAS ankle‐hindfoot score, and FFI score, with a low complication rate at a minimum 15‐month follow‐up.

## DISCLOSURES

The authors (S.S.L.O., R.K.S., I.H.M, C.K.K.K.) declare that they have no known competing financial interests or personal relationships that could have appeared to influence the work reported in this paper.
